# Impact of the COVID-19 pandemic on ovarian cancer diagnosis, treatment, and survival in a Swiss tertiary care hospital - a conclusion five years after the outbreak of the pandemic

**DOI:** 10.3389/fonc.2026.1664719

**Published:** 2026-04-24

**Authors:** Andrea Gmür, Denis Favre, Damaris Erhardt, Hannes Egli, Julian Wampfler, Flurina Saner, Sara Imboden, Michael D. Mueller, Franziska Siegenthaler

**Affiliations:** 1 Department of Obstetrics and Gynecology, Bern University Hospital and University of Bern, Bern, Switzerland; 2 Department of Medical Oncology, Bern University Hospital, Bern, Switzerland

**Keywords:** borderline ovarian tumor (BOT), COVID-19 pandemic, healthcare, oncological outcome, ovarian cancer

## Abstract

**Introduction:**

The outbreak of SARS-CoV-2 in 2020 and the resulting lockdowns had a major impact on general healthcare provision. This study aimed to investigate whether the COVID-19 pandemic affected diagnosis, treatment, and survival of ovarian cancer patients at our institution.

**Methods:**

This retrospective cohort study included patients diagnosed with primary ovarian cancer and borderline ovarian tumor (BOT) at Bern University Hospital between January 2019 and December 2022. Clinical data were collected in a standardized database.

**Results:**

A total of 218 patients were included. Annual distribution of primary ovarian cancer diagnoses was 52 (2019), 32 (2020), 39 (2021), and 57 (2022), while borderline ovarian tumor diagnoses remained stable: 10 (2019, 2020, 2022) and 8 (2021). FIGO stage distribution remained unchanged during the study period (p= .520). Among 131 patients with advanced-stage ovarian cancer, 84% underwent cytoreductive surgery without significant changes over time (p= .838). However, a shift in surgical strategy was observed with interval debulking surgery increasing significantly during the COVID-19 pandemic years (2020 – 2021) (p= .030). Complete cytoreduction was achieved in 89% of cases, remaining constant (p= .355). Chemotherapy was administered to 94% of advanced-stage patients, with no changes during the COVID-19 pandemic years (p= .139). Cox regression analysis revealed no increased risk of recurrence (p= .142) or death (p= .587) for patients diagnosed during the COVID-19 pandemic years (2020–2021) compared to those in 2019 and 2022.

**Conclusion:**

While BOT diagnoses remained stable throughout the COVID-19 pandemic years, primary ovarian cancer diagnoses decreased in 2020 and 2021. No shift in disease stage was observed. However, more patients received interval debulking surgery during the pandemic. Overall, the SARS-CoV-2 outbreak did not negatively affect the oncological outcomes of patients with advanced-stage ovarian cancer at our institution.

## 1 Introduction

On March 11, 2020, the World Health Organization declared COVID-19 a pandemic due to its rapid spread and severity, leading to a global crisis that has profoundly affected health systems worldwide (1). Healthcare resources were redirected to manage COVID-19 patients, resulting in disruptions to routine care, including cancer diagnosis and treatment. The pandemic led to a decrease in cancer diagnoses as well as to a shift in cancer presentation with higher proportions of advanced-stage diagnoses as documented in studies across various malignancies and countries (2–5). Furthermore, significant changes in cancer treatments were observed, including alterations in surgical procedures, chemotherapy regimens, radiotherapy protocols, immunotherapy, and hormonal therapies (6–8).

Ovarian cancer is a highly aggressive gynecologic cancer and a leading cause of cancer-related deaths among women worldwide (9). It is often asymptomatic in early stages, leading to late diagnoses and limited treatment options. Due to its complex presentation, effective management and timely diagnosis are critical for improving patient survival rates. The primary treatment for ovarian cancer is cytoreductive surgery, followed by chemotherapy (10–12). However, the majority of patients with ovarian cancer are diagnosed with advanced disease and a complete macroscopic cytoreduction cannot be achieved in all patients (12). Neoadjuvant chemotherapy followed by interval debulking surgery is a recommended therapeutic strategy for ovarian cancer patients when complete tumor resection appears unachievable (13). Previous studies have demonstrated that ovarian cancer patients experienced disruptions or modifications in various aspects of their care during the COVID-19 pandemic years (14–16).

In Switzerland, the first confirmed COVID-19 case was reported in the southern region on February 25, 2020. Shortly thereafter, substantial restrictions affecting healthcare services were implemented in March 2020, resulting in reduced surgical capacity and limited resources. This study evaluates the impact of the COVID-19 pandemic on ovarian cancer diagnosis, treatment, and outcomes at a Swiss tertiary care hospital. By analyzing changes in diagnostic patterns, treatment strategies, and oncological outcomes over a four-year period, this work aims to provide insights into the adaptability and resilience of cancer care.

Furthermore, five years after the onset of the pandemic, this study seeks to contribute to preparedness strategies by identifying key factors that may help maintain patient safety and continuity of care during future pandemics or other global crises that could again challenge healthcare systems.

## 2 Materials and methods

This retrospective, single-center cohort study included patients with primary diagnosis of ovarian cancer and borderline ovarian tumor at the certified cancer center at the Bern University Hospital, Switzerland, between January 2019 and December 2022. The local ethics committee Bern, Switzerland reviewed the study protocol (Req-2024-01544) and all patients signed written general consent. The primary objective of this study was to assess whether a diagnosis of ovarian cancer during the COVID-19 pandemic years was associated with worse overall survival. Secondary objectives were to evaluate changes in case numbers, disease stage at diagnosis, and treatment strategies (primary versus interval debulking surgery), as well as rates of complete cytoreduction, administration of adjuvant chemotherapy, and the risks of recurrence and death.

### 2.1 COVID restrictions

The Federal Council’s COVID-19 Ordinance of 16 March 2020 stipulated that healthcare facilities such as hospitals and clinics, medical practices and dental practices must refrain from non-urgent (elective) medical interventions and therapies. This rule was in force until 26 April 2020. The cantons and healthcare institutions then decided whether to continue or discontinue interventions and therapies. In our institution at Bern University Hospital, Switzerland, elective, non-urgent operations were canceled or postponed between March and April 2020, as stipulated by the Federal Council. Owing to this limitation, there was no need of restrictions for the operating capacity for patients with cancer.

### 2.2 Clinical data and oncological outcome

Clinicopathological and surgical data for study participants were collected from the institution’s prospectively maintained database. Follow-up data on recurrence and survival were available through standardized databases and follow-up controls. Clinical outcome parameters collected were overall survival, and recurrence-free survival. Recurrence-free survival was defined as time from primary staging surgery to first recurrence or death of any cause. Overall survival was calculated from date of primary staging surgery until death of any cause or until the date of the last follow-up.

### 2.3 Statistical analysis

Statistical analysis was performed using the Statistical Package for Social Sciences (IBM SPSS Statistic version 28.0.1.1). Categorical variables were reported as frequencies and proportions, while continuous variables were reported as means and standard deviations. Formal comparisons were made using Chi-square statistics (χ2) or Fisher’s exact test for categorical variables and T-test or analysis of variance (ANOVA) for continuous variables. Survival analyses were performed using the Kaplan-Meier method and compared using the log-rank test. Cox regression analyses were conducted to assess the relationship between the risk of recurrence and death with other prognostic factors. Statistical significance was defined as a p-value below 0.05.

## 3 Results

Of the 301 patients treated for ovarian cancer or borderline ovarian tumors at our center, 218 provided general consent and were included in the final study cohort; no additional exclusion criteria were applied. **Table 1** summarizes the main clinicopathological characteristics of the study cohort. The mean age was 63 years, and the majority of patients were diagnosed with ovarian cancer (82.6%) and serous histology (68.3%). The incidence of borderline ovarian tumors remained stable throughout the study period, whereas a numerical decrease in ovarian cancer diagnoses was observed in 2020 and 2021. In contrast, the distribution of FIGO stage remained consistent over time.

**Table 1:** Clinicopathological characteristics of the study cohort.

	Total (n = 218)	2019 (n = 62)	2020 (n = 40)	2021 (n = 46)	2022 (n = 67)	p-value
**Mean age**, years ± SD	63.3. ± 15.0	66.1 ± 13.9	63.1 ± 15.0	62.5 ± 14.2	61.4 ± 16.5	.335
Tumor type, n (%)						
- Borderline ovarian tumor - ovarian cancer	38 (17.4) 180 (82.6)	52 (83.9) 10 (16.1)	32 (76.2) 10 (23.8)	39 (83.0) 8 (17.0)	57 (51.8) 10 (14.9)	.669
FIGO stage, n (%)						
- I - II - III - IV	69 (31.7) 15 (6.9) 103 (47.2) 31 (14.2)	16 (25.8) 4 (6.5) 36 (58.1) 6 (9.7)	13 (31.0) 2 (4.8) 22 (52.4) 5 (11.9)	19 (40.4) 3 (6.4) 18 (38.3) 7 (14.9)	21 (31.3) 6 (9.0) 27 (40.3) 13 (19.4)	.520
Histological subtype, n (%)						
- serous - mucinous - endometrioid - clear cell - carcinosarcoma - other - missing	149 (68.3) 28 (12.8) 13 (6.0) 7 (3.2) 6 (2.8) 14 (6.5) 1 (0.5)	45 (72.6) 7 (11.3) 3 (4.8) 3 (4.8) 2 (3.2) 2 (3.2) 0 (0)	29 (69.0) 6 (14.3) 4 (9.5) 0 (0) 1 (2.4) 2 (4.8) 0 (0)	28 (59.6) 7 (14.9) 4 (8.5) 3 (6.4) 2 (4.3) 2 (4.3) 1 (2.1)	47 (70.1) 8 (11.9) 2 (3.0) 1 (1.5) 1 (1.5) 8 (12.0) 0 (0)	.554

n, number; SD, standard deviation; FIGO, Féderation International de Gynecologie et Obstetrique.

Totally, 131 of the study patients were diagnosed with advanced stage (FIGO stage III or IV) ovarian cancer. **Table 2** shows a detailed description of the clinicopathological and treatment characteristics of this subgroup. Overall, 84% of the patients with advanced stage ovarian cancer underwent cytoreductive surgery during primary treatment, without significant alterations over time (p= .838). Reasons for not performing debulking surgery included unresectable disease in 18 patients (13.7%) and frailty in three patients (2.3%). OR-capacity was never a determining factor. However, there was a significant change in the type of primary cytoreductive surgery observed during the pandemic, while 34% of the patients underwent interval debulking surgery in 2019, it was 50% in 2020, 76% in 2021, and 49% in 2022 (p= .030). The proportion of patients who underwent a complete cytoreduction was 89%, remaining constant throughout the study period (p=.355). Mean Aletti surgical complexity score was 5.7 for the subgroup of patients undergoing cytoreductive surgery and showed no significant variation during the study period (p= .241) (17). Furthermore, 94% of patients with advanced-stage ovarian cancer received chemotherapy as their primary treatment, a regimen that remained unaltered during the pandemic (p= .139). In the eight patients who did not receive chemotherapy during primary treatment, the reasons for this decision were frailty in two cases, patient refusal in five cases, and one patient died before the commencement of chemotherapy. Maintenance treatment with PARP-Inhibitors increased significantly throughout the study period (p= .006).

**Table 2:** Clinicopathological and treatment characteristics of the subgroup of patients with advanced stage ovarian cancer.

	Total (n = 131)	2019 (n = 40)	2020 (n = 26)	2021 (n = 25)	2022 (n = 40)	p-value
Mean age, years ± SD	66.3 ± 12.4	67.3 ± 12.8	64.5 ± 12.5	67.3 ± 12.5	65.8 ± 12.3	0.806
FIGO stage, n (%)						
- III	100 (76.3)	34 (85.0)	21 (80.8)	18 (72.0)	27 (67.5)	
- IV	31 (23.7)	6 (15.0)	5 (19.2)	7 (28.0)	13 (32.5)	0.269
Histological subtype, n (%)						
- high-grade serous	101 (77.1)	27 (67.5)	19 (73.1)	20 (80.0)	35 (87.5)	
- low-grade serous	11 (8.4)	4 (10.0)	4 (15.4)	1 (4.0)	2 (5.0)	
- mucinous	5 (3.8)	4 (10.0)	0 (0)	1 (4.0)	0 (0)	
- endometrioid	2 (1.5)	1 (2.5)	1 (3.8)	0 (0)	0 (0)	
- clear cell	2 (1.5)	1 (2.5)	0 (0)	1 (4.0)	0 (0)	
- carcinosarcoma	6 (4.6)	2 (5.0)	1 (3.8)	2 (8.0)	1 (2.5)	
- other	4 (3.1)	1 (2.5)	1 (3.8)	0 (0)	2 (5.0)	0.532
CA 125 before surgery, U/ml ± SD	1275.7 ± 1956.1	1171.2 ± 1741.0	781.6 ± 1325.3	1191.7 ± 1791.0	1748.7 ± 2489.9	0.26
Ascites, more than 500ml, n (%)	46 (35.1)	13 (32.5)	8 (30.8)	5 (20.0)	20 (50.0)	0.096
Cytoreductive surgery for primary treatment, n (%)	110 (84.0)	32 (80.0)	22 (84.6)	21 (84.0)	35 (87.5)	0.838
Type of cytoreductive surgery, n (%)						
- Primary debulking surgery	55 (42.0)	21 (52.5)	11 (42.3)	5 (20.0)	18 (45.0)	
- Interval debulking surgery	55 (42.0)	11 (27.5)	11 (42.3)	16 (64.0)	17 (42.5)	
- No surgery	21 (16.0)	8 (20.0)	4 (13.8)	4 (16.0)	5 (12.5)	0.03
Mean Aletti surgical complexity score ± SD	5.7 ± 2.5	6.1 ± 2.3	5.7 ± 3.0	4.7 ± 1.7	5.8 ± 2.7	0.241
Residual disease after primary surgery, n (%)	15 (11.5)	6 (15.0)	3 (11.5)	0 (0)	6 (15.0)	0.242
Chemotherapy for primary treatment, n (%)	123 (93.9)	35 (87.5)	24 (92.3)	25 (100)	39 (97.5)	0.139
Maintenance therapy after primary treatment, n (%)						
- None	62 (47.3)	25 (62.5)	18 (69.2)	9 (36.0)	10 (25.0)	
- Bevacizumab	24 (18.3)	6 (15.0)	3 (11.5)	7 (28.0)	8 (20.0)	
- PARP-Inhibitor	23 (17.6)	8 (20.0)	3 (11.5)	3 (12.0)	9 (22.5)	
- Bevacizumab plus PARP-Inhibitor	16 (12.2)	0 (0)	2 (7.7)	5 (20.0)	9 (22.5)	
- Other	6 (4.6)	1 (2.5)	0 (0)	1 (4.0)	4 (10.0)	0.006

n, number; SD, standard deviation; FIGO, Féderation International de Gynecologie et Obstetrique; BRCA, BReast Cancer; CA 125, Cancer-Antigen 125; PARP, poly ADP ribose polymeras.

**Figure 1:**
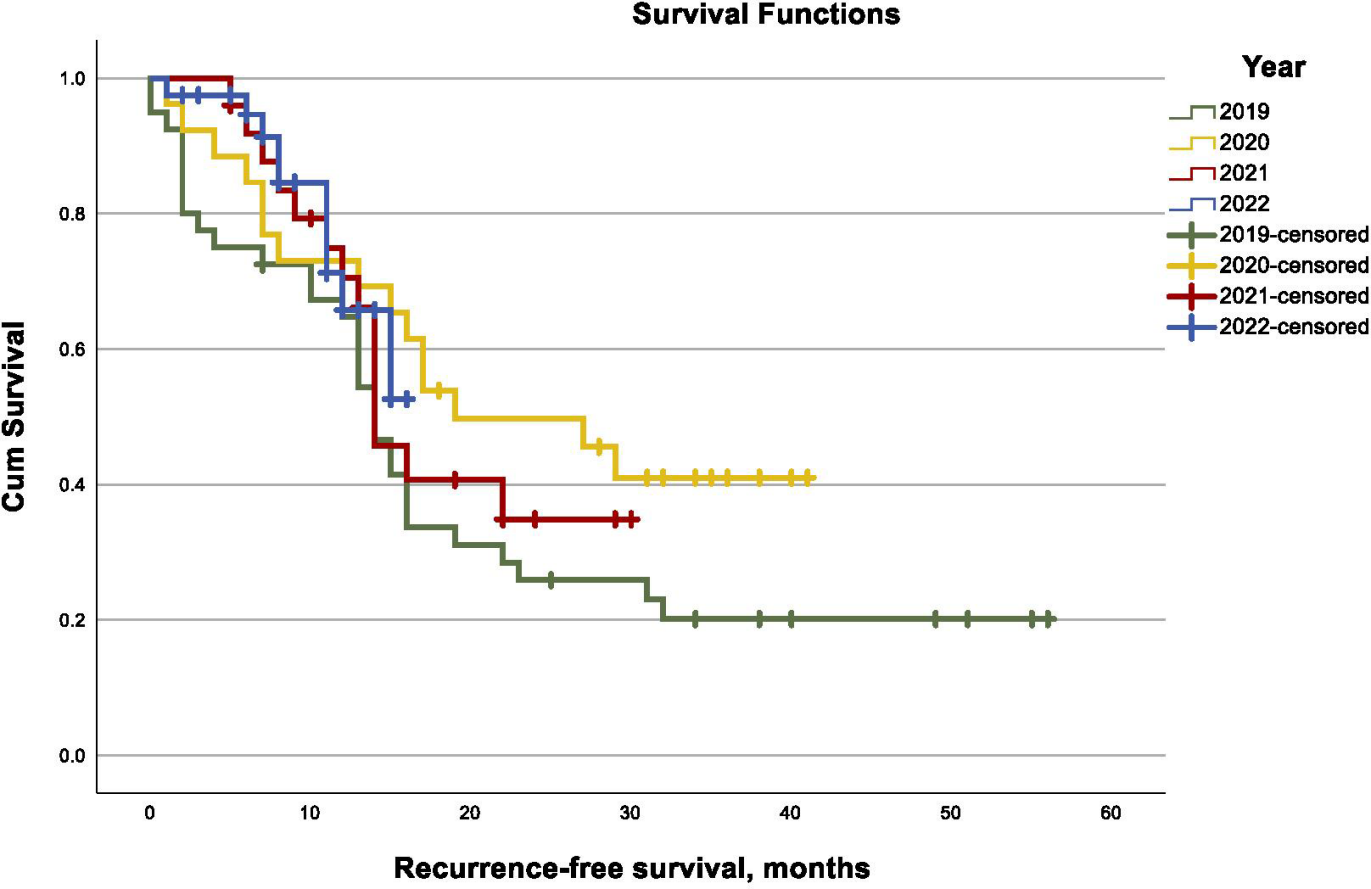
Kaplan–Meier curves of recurrence-free survival in advanced-stage ovarian cancer by year (2019–2022).

**Figure 2:**
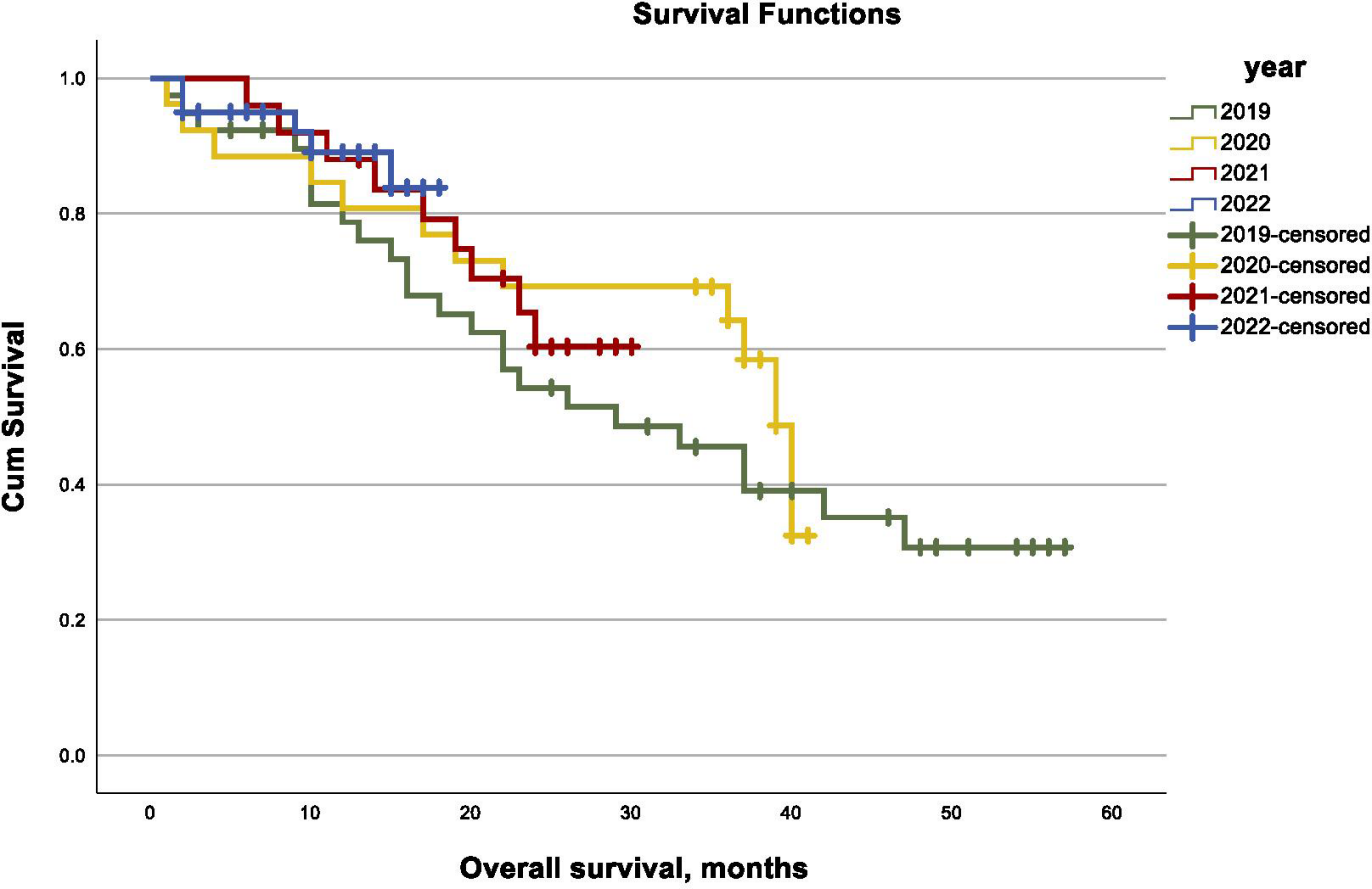
Kaplan–Meier curves of overall survival in advanced-stage ovarian cancer by year (2019–2022).

Mean follow-up was 29.2 (95% CI 26.1 – 32.2) months for patients with advanced stage ovarian cancer. Of these patients, totally 65 (49.6%) experienced at least one recurrence and 50 (38.2%) died. Mean recurrence-free survival in this subgroup was 25.8 (95% CI 21.6 – 30.0) months with no significant difference between the study years (log-rank, p= .216, **Figure 1**). Mean overall survival was 35.5 (95% CI 31.4 – 39.7) months for patients with advanced ovarian cancer with a consistent distribution over the course of the study years (log-rank, p= .582, **Figure 2**). In univariable Cox regression analysis, diagnosis of advanced stage ovarian cancer during the pandemic years 2020 and 2021 was not associated with a higher risk of recurrence (HR 1.4, 95% CI 0.882 – 2.405) or death (HR 1.2, 95% CI 0.658 – 2.098, p= .587) compared to patients diagnosed in 2019 or in 2022. Also, in multivariable Cox regression analysis including stage, residual disease after surgery and PARP-inhibitor maintenance, there was no increased risk of recurrence (HR 1.5, 95% CI 0.893 – 2.745, p=0.117) or death (HR 1.4, 95%-CI 0.694 – 2.764, p= 0.356) for patients diagnosed with advanced stage ovarian cancer during the COVID-19 pandemic years.

## 4 Discussion

This study evaluated the impact of the COVID-19 pandemic on the diagnosis, treatment, and outcomes of ovarian cancer at our tertiary cancer center, Bern University Hospital, Switzerland. At our center, we observed a decrease in the number of patients with primary ovarian cancer diagnoses during the COVID-19 pandemic years, 2020 and 2021, compared with the pre-pandemic (2019) and post-pandemic (2022) years. Psychological factors and potential patient hesitancy to seek medical care during the pandemic may have influenced presentation patterns (14). However, such aspects cannot be fully captured within a retrospective study design and should therefore be interpreted cautiously. Similar findings have been reported by several European gynecologic oncology centers, describing pandemic-related delays in cancer diagnosis and disruptions to oncologic care pathways (2, 3, 16, 18). In contrast, FIGO stage distribution remained stable throughout the study period, and no stage migration toward more advanced-stage disease was observed. This finding differs from several reports indicating that patients diagnosed with various cancers during the pandemic presented at more advanced stages (2–4), including one study focusing on ovarian cancer (19).

In this study, the vast majority of patients underwent cytoreductive surgery as primary treatment during the study period, and this did not change over time. Nevertheless, the type of primary cytoreductive surgery shifted significantly toward interval debulking during the COVID-19 pandemic years, with a peak in 2021. Although surgical capacity for oncologic patients was formally maintained at our institution, this observation may reflect changes in clinical decision-making or organizational constraints during the pandemic years. As data on the time from diagnosis to surgery were not available, potential delays or underlying causal mechanisms cannot be fully assessed. These changes in surgical practice are consistent with a multicenter international prospective cohort study by Fotopoulou et al. which reported that one in five gynecologic cancer patients experienced adjustments to standard care during the COVID-19 pandemic (16). Alterations in surgical treatment, including delays and cancellations for surgery in patients with gynecological cancer have also been described by other cancer centers (20–22). In our cohort, the proportion of patients achieving complete cytoreduction remained consistently high, indicating sustained surgical quality, further supported by stable Aletti surgical complexity scores throughout the study period. Analogous observations were made in the field of oncological treatment, wherein the proportion of patients with advanced-stage ovarian cancer receiving chemotherapy as a primary treatment remained constant over the years. The increasing use of PARP inhibitor maintenance observed in our cohort likely reflects the evolving implementation of targeted therapies and expanding guideline recommendations during the study period, rather than pandemic-related treatment changes (23–25).

In our cohort, oncological outcomes in advanced-stage ovarian cancer did not appear to worsen during the COVID-19 pandemic years, although the findings should be interpreted as observational rather than definitive. Patients with advanced-stage ovarian cancer diagnosed in 2020 and 2021 did not show a higher risk of recurrence or death compared with those diagnosed in 2019 or 2022. These results are consistent with previously published data (26). The observed stability in surgical and oncological outcomes may reflect characteristics of our tertiary care setting, including established triaging strategies, close multidisciplinary collaboration, and consistent surgical expertise, which supported continuity of care during the pandemic. According to our findings, operating room capacity was not a limiting factor, partly due to the postponement of elective procedures, allowing prioritization of oncologic surgery.

To the best of our knowledge, this is the first study to assess the impact of the COVID-19 pandemic on ovarian cancer in Switzerland. Another major strength of this study includes the relatively long follow-up. The main limitation of this retrospective study is the potential for selection and information bias due to reliance on available clinical records and consented participants. The relatively small sample size, particularly among patients with borderline ovarian tumors, limits statistical power, and the lack of external validation may restrict the generalizability of our findings. In addition, mortality directly attributable to COVID-19 infection was not specifically analyzed within this cohort.

## 5 Conclusion

The COVID-19 pandemic led to a decrease in ovarian cancer diagnoses and a shift toward increased interval debulking surgeries at our institution, but did not negatively affect oncological outcome of patients diagnosed with advanced stage ovarian cancer. Through the analysis of changes in diagnostic patterns, treatment approaches, and oncological outcomes over this four-year period, these findings highlight the resilience of our institution’s healthcare service during a global health crisis. Given the ongoing global instability and the potential for future crises with resource constraints, our established approach may remain crucial in ensuring continued high-quality patient care.

## Data Availability

The original contributions presented in the study are included in the article/supplementary material. Further inquiries can be directed to the corresponding author.
